# Congenital giant megaureter prenatally diagnosed: A case report

**DOI:** 10.1016/j.eucr.2022.102244

**Published:** 2022-09-26

**Authors:** Souha Laarif, Aida Daïb, Rabiaa Ben Abdallah, Cyrine Saadi, Youssef Hellal, Nejib Kaabar

**Affiliations:** Pediatric Surgery Department, Habib Thameur Hospital, Tunis, Tunisia

## Abstract

Congenital giant megaureter (CGM) is uncommon in pediatic population, defined as congenital localized or total dilatation of the ureter to over 10 times the normal diameter with a normal bladder. Herein, we reported an entirely dilated CGM presented as neonatal bowel obstruction in a newborn baby.

Our experience has suggested that CGM should be considered as a differential diagnosis of abdominal distension and occlusive syndrome.

## 1 Introduction

Congenital giant megaureter (CGM) is a very rare condition defined as congenital localized or total dilatation of the ureter to over 10 times the normal diameter with a normal bladder.^1^ Primary or congenital mega-ureter is a common cause of fetal hydronephrosis discovered prenatally. It is related to a functional obstacle at the uretero-bladder junction.^2^ Diagnosis and management are usually made very early in childhood.

Herein, we reported an entirely dilated CGM presented as neonatal bowel obstruction. In addition, we reviewed the epidemiology, pathogenesis, diagnosis, and therapies of this rare condition by analyzing previously reported case.

## 2 Case presentation

**Fig. 1:**
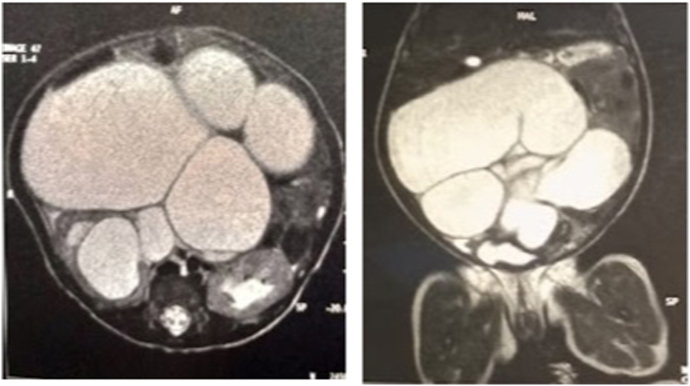
MRI cross-section in 1–1 showing bilateral hydronephrosis and right geant megaureter and ipsilateral renal parenchyma thinning. Coronal section in^1^,^2^ showing an important ureter dilatation.

A 1-day-old full-term girl (3.6 kg), with a prenatal ultrasonographic and fœtal magnetic resonance imaging (MRI) diagnosis of “intestinal dilatation” since the end of the fifth month of gestation, with bilateral kidney dilatation and right-sided renal parenchyma thinning (Fig. 1), was transferred to our pediatric surgery department for severe abdominal distension.

**Fig. 2:**
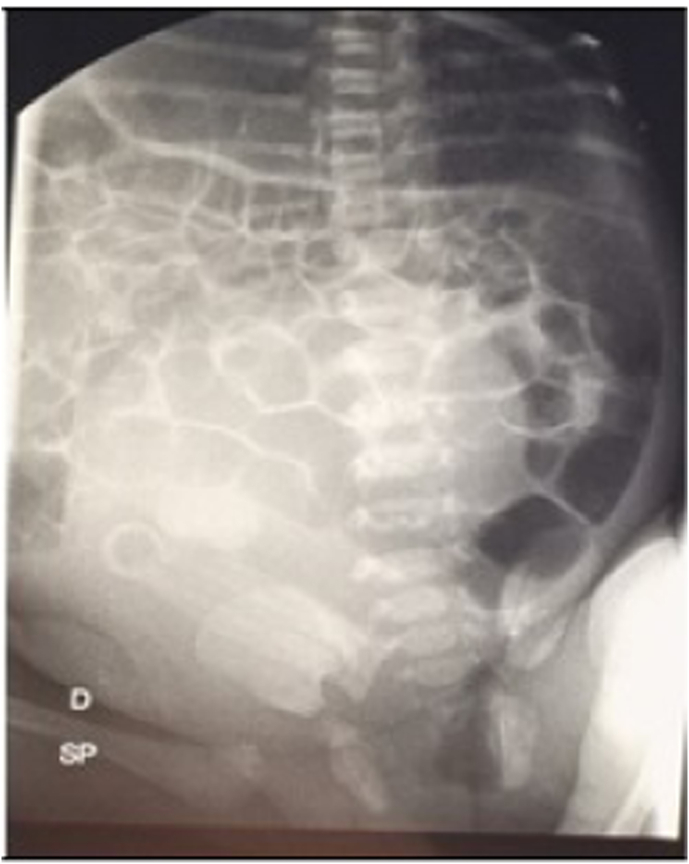
a mass completely filling the lower abdominal cavity visible on abdominal x-ray.

The first passage of meconium had been regular, and then she developed a bowel obstruction. The renal biochemistry and urinalysis findings were normal. A plain abdominal x-ray (Fig. 2) showed a mass that occupied the entire right side of the abdomen and in its lower part, crossed the midline almost filling the lower abdominal cavity; the small intestine and colon were displaced upward and to the left.

An ultrasound scan showed an important small bowel dilatation; with bilateral renal dilatation. Because of abdominal distension and unresponsive bowel obstruction to nursing, it was decided to explore the child with a laparotomy. At laparotomy, we found a huge tortuous right megaureter that represses all the intestinal tracts backward. A cutaneous ureterostomy was performed. The postoperative period was uneventful. A MAG-III scan was performed and had shown a very low cortical caption in the right kidney with an estimated function of 8%. Due to the occurrence of two urinary tract infections, it was decided to perform a right ureteronephrectomy. The baby is now a 10-year-old healthy child, with a normal left renal function on DMSA scan and a normal bladder and urethra on voiding cystourethrography.

## 3 Discussion

The first CGM was published by Chaterjee SK in 1964.^1^ Since then, a small number of patients with CGM have been reported. Among cases with CGM that were published, the sex ratio was approximately 1:1. Unlike congenital megaureters which could be seen bilaterally in about 20% of cases,^3^ all the patients reported with CGM were unilateral. In our case, the CGM was on the left side.

The diagnosis of CGM was habitually based on history, physical examinations, and imaging examinations.^3^ In the present case, the diagnosis of CGM with the left giant ureter imitating Hirschsprung disease was made according to physical examination, imaging examination, and operative findings.

Ultrasound is an effective method of assessing the fetal urinary tract and can deliver the correct diagnosis in the vast majority of cases.^2^ Nevertheless, some technical limitations may hinder the use of ultrasound. Then, when ultrasound is inconclusive, MRI provides additional important information. Like in our case, where MRI was necessary for further findings. In addition, magnetic resonance urography (MRU) combined with urography can clearly show the features of the megaureter, particularly the extent of the dilated ureter and renal pelvis, and the site of the narrow segment.^2^ Therefore, MRU may be a good choice for patients in early life.

The current case, although misdiagnosed, was detected prenatally. At birth, abdominal distension and bowel obstruction suggested immediate surgical treatment, without prior complete urological examination which is moreover mandatory to exclude associated anomalies and decide the appropriate surgical procedure.

The treatment of congenital megaureter is controversial. Some authors proposed early surgical intervention, whereas others suggested temporary conservative treatment for most patients.^3^ Compared to congenital megaureter, the treatment of CGM is quite specific.^3^ Ureteroureterostomy after excision of the dilated segment or ureteral reimplantation is efficient for patients with segmental dilation and a preserved renal function^4^; although, for patients with a dilated whole ureter and poor renal function, ureteronephrectomy may be a good choice.^3^ On the other hand; age can also determine the choice of surgical intervention. In infants older than 1 year, the preferred procedure for the majority was ureteral reimplantation with or without ureteral tapering. Some authors, however, agreed that reimplantation of a grossly dilated ureter into a relatively small infant bladder could be a difficult operation in babies younger than 1 year of age, and some other temporary or definitive options were discussed.^5^ These included endoureterotomy, cutaneous ureterostomy, temporary double-J stenting, and endoscopic balloon dilation.^5^ In case of emergency intervention, when faced with a septic child with an obstructed infected system that is not responding to intravenous antibiotics, the most author preferred percutaneous nephrostomy or cutaneous ureterostomy over double-J stenting.^5^

## 4 Conclusion

Cases of CGM have been reported in newborn infants or there was a history of a mass since birth in other cases of CGM(4). Our experience has suggested that, in the differential diagnosis of abdominal distension and occlusive syndrome, CGM should also be considered.
